# Crystal structure of human endothelin ET_B_ receptor in complex with peptide inverse agonist IRL2500

**DOI:** 10.1038/s42003-019-0482-7

**Published:** 2019-06-21

**Authors:** Chisae Nagiri, Wataru Shihoya, Asuka Inoue, Francois Marie Ngako Kadji, Junken Aoki, Osamu Nureki

**Affiliations:** 1 Department of Biological Sciences, Graduate School of Science, The University of Tokyo, Bunkyo-ku, Tokyo, 113-0033 Japan; 2 Graduate School of Pharmaceutical Sciences, Tohoku University, 6-3, Aoba, Aramaki, Aoba-ku, Sendai, 980-8578 Miyagi Japan

**Keywords:** X-ray crystallography, Cardiovascular biology

## Abstract

Endothelin receptors (ET_A_ and ET_B_) are G-protein-coupled receptors activated by endothelin-1 and are involved in blood pressure regulation. IRL2500 is a peptide-mimetic of the C-terminal tripeptide of endothelin-1, and has been characterized as a potent ET_B_-selective antagonist, which has preventive effects against brain edema. Here, we report the crystal structure of the human ET_B_ receptor in complex with IRL2500 at 2.7 Å-resolution. The structure revealed the different binding modes between IRL2500 and endothelin-1, and provides structural insights into its ET_B_-selectivity. Notably, the biphenyl group of IRL2500 penetrates into the transmembrane core proximal to D^2.50^, thus stabilizing the inactive conformation. Using the newly-established constitutively active mutant, we clearly demonstrate that IRL2500 functions as an inverse agonist for the ET_B_ receptor. The current findings will expand the chemical space of ETR antagonists and facilitate the design of inverse agonists for other class A GPCRs.

## Abstract (web summary)

Chisae Nagiri and Wataru Shihoya report the crystal structure of the human endothelin receptor ET_B_ in complex with an ET_B_ antagonist IRL2500, suggesting different binding modes between IRL2500 and endothelin-1, ET_B_’s endogenous ligand. This study provides structural insights into the specificity of ET_B_ interaction with its ligands.

## Introduction

Endothelin receptors (ETRs) are G-protein-coupled receptors (GPCR) activated by vasoactive peptide, endothelins^1^. Two ETR subtypes (ET_A_ and ET_B_) are widely expressed in the vascular endothelium, brain, and other circulatory organs^2,3^. Endothelin-1 (ET-1) activates the both ETRs with sub-nanomolar affinities. The activation of the ET_A_ receptor leads to potent and long-lasting vasoconstriction, whereas that of the ET_B_ receptor induces nitric oxide-mediated vasorelaxation. Therefore, the up-regulation of ET-1 is related to circulatory-system diseases, including pulmonary arterial hypertension (PAH)^4–7^. Moreover, the autocrine and paracrine signaling functions of ET-1 through the ET_A_ receptor play a critical role in tumor growth and survival^8^. Thus, ETR antagonists have been developed for the treatment of circulatory-system diseases and cancers^6,7^. Bosentan is the first orally-active ETR antagonist^9,10^, and is used to treat PAH. The ET_B_ receptor is the prominent ETR subtype in the brain, with high expression levels in astrocytes^11^. Stimulation of the ET_B_ receptor modulates astrocytic responses, indicating its important roles in regulating astrocytic functions^12^. The up-regulation of the astrocytic ET_B_ receptor by ET-1 increases the vascular permeability and reduces the AQP4 levels, thereby aggravating vasogenic brain edema^11^. The application of ET_B_-selective antagonists may provide preventive effects against brain edema in the acute phase of brain insults^13–16^.

To date, most ETR antagonists have been developed based on bosentan^17,18^. The ETR antagonists that have been developed till now are mostly N-heterocyclic sulfonamides with similar structures and molecular weights, and non-sulfonamide antagonists (atrasentan, ambrisentan, darusentan, and enrasentan) still retain high similarities with each other and with the sulfonamides^6^. Since the ETR antagonists are chemically very similar^7^, and the expanded chemical space should be exploited. IRL2500 is a peptide-mimetic ETR antagonist developed based on the partial region of ET-1^19^, not on bosentan. IRL2500 has been characterized as an ET_B_-selective antagonist with an IC_50_ value of 1.2 nM^20^, which shows higher affinity than that of bosentan. In an animal model, the intracerebroventricular administration of IRL2500 attenuated the cold injury-mediated brain edema and disruption of the blood–brain barrier, indicating the neuroprotective effect of IRL2500^14,15^. Clarification of the IRL2500 binding mode would facilitate the expansion of the chemical space of ET agents.

We previously reported the crystal structures of the ET_B_ receptor bound to ET-1^21^ and bosentan^22^; however, both the binding mode and ET_B_-selectivity of IRL2500 remained to be elucidated. Here, we present the crystal structure of the ET_B_ receptor in complex with IRL2500. This structure revealed the unique binding mode of IRL2500, which differs from those of ET-1 and bosentan. Structure-guided functional analyses clearly demonstrate that IRL2500 functions as an inverse agonist for the ET_B_ receptor, and thus will provide the basis for the design of inverse agonists for other class A GPCRs.

## Results

### Overall structure

**Fig. 1:**
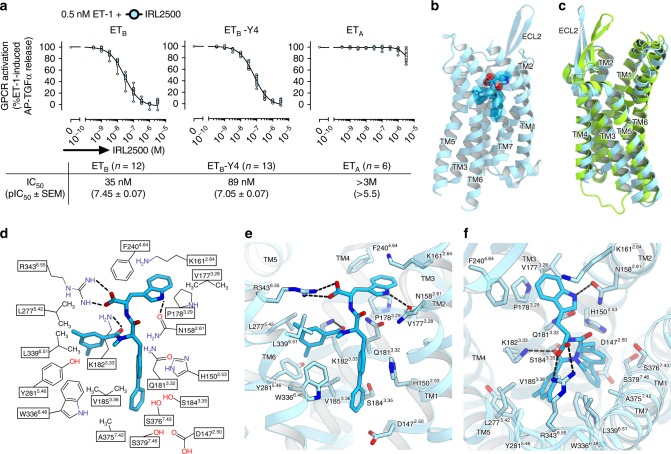
Overall structure of the IRL2500-bound ET_B_ receptor. **a** The effect of IRL2500 on the ET-1 (0.5 nM)-induced release of AP-TGFα in HEK293 cells expressing the endothelin receptors. For each experiment, the AP-TGFα release response in the absence of IRL2500 is set at 100%. Concentration-response data are displayed as means ± SEM (standard error of the mean) from six to thirteen independent experiments and the pIC_50_ values are from the indicated number of independent experiments. **b** The overall structure of the IRL2500-bound ET_B_ receptor. The receptor is shown as a sky blue ribbon model. IRL2500 is shown as a deep sky blue stick model with a transparent surface model. **c** Superimposition of the IRL2500-bound and ligand-free ET_B_ structures (PDB code: 5GLI), colored sky blue and light green, respectively. **d** Schematic representation of the interactions between ET_B_ and IRL2500 within 4.5 Å. The dashed lines show hydrogen bonds. **e**, **f** Binding pocket for IRL2500, viewed from the membrane plane (**e**) and the extracellular side (**f**). The receptor is shown in a sky blue ribbon representation. IRL2500 and receptor residues involved in ligand binding are shown as sticks, colored deep sky blue and sky blue, respectively. The dashed lines show hydrogen bonds

For crystallization, we used the previously established, thermostabilized ET_B_ receptor (ET_B_-Y4)^22,23^. The IC_50_ value of IRL2500 for ET_B_-Y4 was similar to that for the wild type receptor in the TGFα shedding assay^24^ (Fig. 1a), suggesting that the thermostabilizing mutations minimally affect the IRL2500 binding. In contrast, the IC_50_ value of IRL2500 for the ET_A_ receptor is over 3 μM (Fig. 1a), indicating that IRL2500 has over 100-fold ET_B_-selectivity, consistent with the previous pharmacological analysis^20^. To facilitate crystallization, we replaced the third intracellular loop (ICL3) of the receptor with minimal T4 Lysozyme^25^ (ET_B_-Y4-mT4L). Using in meso crystallization^26^, we obtained crystals of ET_B_-Y4-mT4L in complex with IRL2500 (Supplementary Fig. 1a, b). In total, 58 datasets were collected and merged by the data processing system KAMO^27^. Eventually, we determined the ET_B_ structure in complex with IRL2500 at 2.7 Å resolution, by molecular replacement using the antagonist-bound ET_B_ structure (PDB code: 5X93) (Table 1).

**Table 1:** Data collection and refinement statistics

	IRL2500-ET_B_
**Data collection**	
Space group	*I4*22
Cell dimensions	
* a*, *b*, *c* (Å)	110.0, 110.0, 291.7
*α*, *β*, *γ* (°)	90, 90, 90
Resolution (Å)^a^	43.88–2.70 (2.797–2.70)
*R*_meas_^a^	0.295 (3.946)
〈*I*/σ(*I*)〉^a^	10.74 (1.08)
CC_1/2_^a^	0.995 (0.798)
Completeness (%)^a^	99.66 (99.35)
Redundancy^a^	19.3 (19.6)
**Refinement**	
Resolution (Å)	43.88–2.70
No. reflections	25,069
*R*_work_/*R*_free_	0.2216/0.2653
No. atoms	
Protein	3258
Ligand	43
Water/ion/lipid	240
Averaged *B*-factors (Å^2^)	
Protein	83.41
Ligand	51.4
Water/ion/lipid	94.45
R.M.S. deviations from ideal	
Bond lengths (Å)	0.003
Bond angles (°)	0.65
Ramachandran plot	
Favored (%)	99.26
Allowed (%)	0.74
Outlier (%)	0

^a^Values in parentheses are for highest-resolution shell

The overall structure consists of the canonical 7 transmembrane helices (TM), the amphipathic helix 8 at the C-terminus (H8), and two antiparallel β-strands in the extracellular loop 2 (ECL2), as in the previously determined ET_B_ structures (Fig. 1b). The IRL2500-bound structure is similar to the bosentan-bound structure, rather than the ET-1-bound structure (R.M.S.D. values for Cα atoms = 1.34 and 1.95 Å, respectively), reflecting the inactive conformation. We observed a remarkable difference in the conformation of ECL2. The β strands are opened up by 9 Å, as compared with those in the ligand-free structure (Fig. 1c and Supplementary Fig. 2a), and are the widest among the peptide-activated class A GPCRs (Supplementary Fig. 2b). This conformation is facilitated by the crystal packing (Supplementary Fig. 2c), and is not a consequence of IRL2500 binding. This structural feature indicates the ECL2 is highly flexible in the inactive conformation of the ET_B_ receptor, to capture the large peptide ligand endothelin.

### IRL2500 binding site

We first describe the IRL2500 binding mode. IRL2500 consists of a tryptophan, a biphenyl group and a 3,5-dimethylbenzoyl group^19^. The biphenyl group forms a peptide bond with the tryptophan, and a peptoid bond with the dimethylbenzoyl group (Fig. 1d). IRL2500 binds to the transmembrane binding cleft exposed to the extracellular side, with a clear electron density (Supplementary Fig. 3a, b). The carboxylate group of the tryptophan moiety in IRL2500 forms salt bridges with R343^6.55^ (superscripts indicate Ballesteros–Weinstein numbers^28^) (Fig. 1e, f). The tryptophan side chain of IRL2500 forms a hydrogen bond with the carbonyl group of the N158^2.61^ side chain and a cation–π interaction with the K161^2.64^ side chain, The tryptophan also forms extensive van der Waals interactions with V177^3.28^, P178^3.29^, and F240^4.64^. The dimethylbenzoyl group of IRL2500 forms van der Waals interactions with the hydrophobic pocket, and is surrounded by V185^3.36^, L277^5.42^, Y281^5.46^, W336^6.48^ and L339^6.51^. The biphenyl group penetrates deeply into the receptor core proximal to D147^2.50^, and forms van der Waals interactions with D147^2.50^, H150^2.53^, W336^6.48^, and S376^7.43^. Overall, the carboxylate of IRL2500 is specifically recognized by the positively charged residues of the ET_B_ receptor, and the other moieties fill the space within the transmembrane binding pocket.

**Fig. 2:**
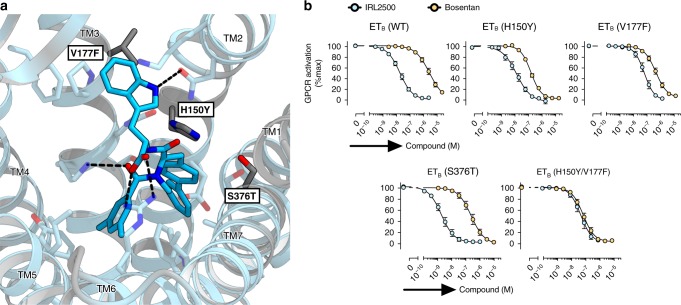
Conservation of the IRL2500 binding site. **a** Sequence conservation of the IRL2500 binding site between ET_A_ and ET_B_, mapped onto the IRL2500-bound structure. Conserved and non-conserved residues are colored sky blue and gray, respectively. The receptor residues involved in IRL2500 binding are shown as sticks. The dashed lines show hydrogen bonds. **b** Effects of IRL2500 and bosentan on the ET-1 (0.5 nM)-induced release of AP-TGFα in HEK293 cells expressing the mutant ET_B_ receptors. For each experiment, the AP-TGFα release response in the absence of IRL2500 is set at 100%. Data are displayed as means ± SEM (standard error of the mean) from four to six independent experiments

To elucidate the structural basis for the ET_B_-selectivity of IRL2500, we compared the residues constituting the IRL2500 binding site between the ET_B_ and ET_A_ receptors (Fig. 2a and Supplementary Fig. 4). Most of the residues are conserved, while H150^2.53^, V177^3.28^, and S376^7.43^ are substituted for the bulkier residues Y129^2.53^, F161^3.28^, and T359^7.43^ in the ET_A_ receptor, respectively. These substitutions may cause steric clashes with the aromatic groups of IRL2500 and reduce its affinity. To investigate this hypothesis, we measured the IC_50_ values of IRL2500 for the H150Y, V177F, and S376T ET_B_ receptor mutants. These mutants showed similar responses for ET-1 in the TGFα shedding assay (Supplementary Fig. 5a). The H150Y mutant showed a similar response for IRL2500, and the S376T mutant showed a 3-fold increased potency (that is, 3-fold smaller IC_50_value) of IRL2500. Only V177F showed a 6-fold decreased potency of IRL2500 (Fig. 2b and Table 2), suggesting that the F161^3.28^ in the ET_A_ receptor sterically clashes with the tryptophan moiety of IRL2500. Moreover, the H150Y/V177F double mutant showed the further decreased potency of IRL2500 by about 1.5-fold as compared with that of the V177F mutant only (Fig. 2b and Table 2), suggesting that the two residues have some cooperativity in the ET_A_ receptor. Notably, these mutations did not reduce (actually increased) the potency of the non-selective antagonist bosentan, showing that the two residues (H150Y and V177F) were selective to the IRL2500 inhibition. Taken together, Y129^2.53^ and F161^3.28^ in the ET_A_ receptor may specifically clash with IRL2500, partially accounting for its ET_B_-selectivity. This is consistent with the results of the previous study, in which the replacement of the tryptophan moiety with the smaller moiety valine weakened its ET_B_-selectivity^29^.

**Table 2:** Pharmacological characterization of mutant ET_B_ receptors

		ET_B-_-WT	H150Y	V177F	S376T	H150/V177F
	*n*	6	4	4	4	4
ET-1	EC_50_ (nM)	0.14	0.090	0.093	0.16	0.11
	pEC_50_ (mean ± SEM)	9.85 ± 0.07	10.05 ± 0.10	10.03 ± 0.11	9.79 ± 0.04	9.97 ± 0.08
IRL2500	IC_50_ (nM)	21	18	130	6.1	180
	pIC_50_ (mean ± SEM)	7.67 ± 0.09	7.74 ± 0.15	6.87 ± 0.13	8.22 ± 0.15	6.75 ± 0.15
	*K*_B_ (nM)	2.4	0.78	7.8	0.63	12
	p*K*_B_ (mean ± SEM)	8.62 ± 0.07	9.11 ± 0.12	8.11 ± 0.07	9.20 ± 0.08	7.93 ± 0.14
	∆*K*_B_	1	3.0	0.30	4.0	0.20
	∆p*K*_B_ (mean ± SEM)	0	0.47 ± 0.11	−0.53 ± 0.06	0.60 ± 0.03	-0.70 ± 0.14
Bosentan	IC_50_ (nM)	3,300	270	1100	680	270
	pIC_50_ (mean ± SEM)	5.48 ± 0.11	6.57 ± 0.05	5.97 ± 0.11	6.17 ± 0.16	6.56 ± 0.11
	*K*_B_ (nM)	400	12	70	71	17
	p*K*_B_ (mean ± SEM)	6.39 ± 0.09	7.92 ± 0.13	7.16 ± 0.05	7.15 ± 0.10	7.78 ± 0.02
	∆*K*_B_	1	35	6.1	5.8	26
	∆p*K*_B_ (mean ± SEM)	0	1.55 ± 0.12	0.79 ± 0.08	0.76 ± 0.11	1.41 ± 0.06

### Comparison of the binding modes of IRL2500, ET-1, and bosentan

**Fig. 3:**
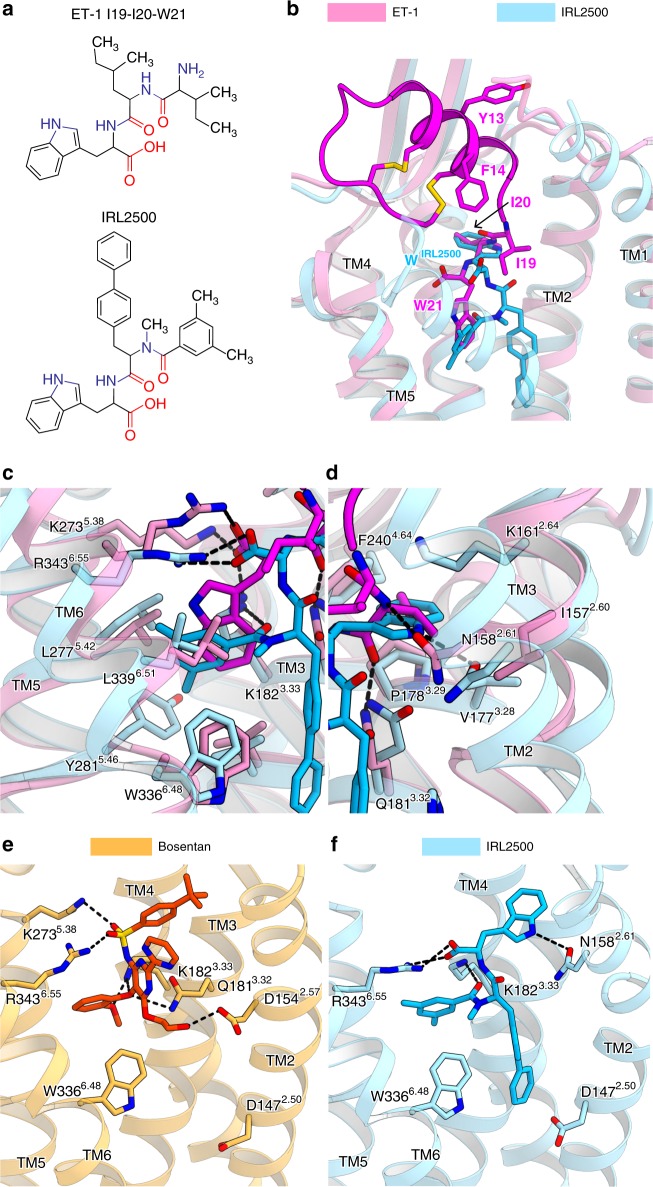
Comparison of binding modes of IRL2500, ET-1, and bosentan. **a** Chemical structures of IRL2500 and the C-terminal tripeptide of ET-1. **b** Superimposition of the IRL2500- and ET-1-bound ET_B_ receptors (PDB code: 5GLH). The ET-1- and IRL2500-bound receptors are shown as pink and sky blue ribbons, respectively. IRL2500 is shown as a stick model. ET-1 is shown as a magenta ribbon with stick models of the peptide residues (Y13, F14, I19, I20, and W21). **c**, **d** The residues interacting with both IRL2500 and ET-1 are shown as sticks. **e**, **f** Binding pockets for bosentan (**e**) and IRL2500 (**f**), viewed from the membrane plane. The bosentan-bound receptor (PDB code: 5XPR) is shown as a thin orange ribbon model. The residues involved in bosentan binding (D154^2.57^, Q181^3.32^, K182^3.33^, K273^5.38^, W336^6.48^ and R343^6.55^) and D147^2.50^ are shown as sticks. Bosentan is shown as an orange stick model. IRL2500 and the IRL2500-bound receptor are colored as in panel (**a**). The residues involved in IRL2500 binding (N158^2.61^, K182^3.33^, R343^6.55^, D147^2.50^ and W336^6.48^) are shown as sticks

IRL2500 is designed to mimic the F14, I19, I20, and W21 residues in ET-1, which play critical roles in ligand binding to the ET_B_ receptor^19^. The tryptophan and dimethylbenzoyl groups of IRL2500 seem to be equivalent to W21 and I19 in ET-1, respectively, while the biphenyl group of IRL2500 seems to be equivalent to F14 and I20 of ET-1 (Fig. 3a). However, a comparison between the IRL2500 and ET-1 binding revealed an unexpected difference in their binding interactions (Fig. 3b). The carboxylate of the tryptophan in IRL2500 superimposes well with that of W21 in ET-1, and is coordinated by similar positively charged residues (Fig. 3c). The tryptophan of IRL2500 does not superimpose with the W21 of ET-1, but do with the I20. Instead, the dimethylbenzoyl group of IRL2500 superimposes with the W21. Nevertheless, these hydrophobic moieties of IRL2500 and ET-1 form comparable hydrophobic interactions (Fig. 3c, d). In contrast, the biphenyl group of IRL2500 penetrates into the receptor core, in an opposite manner to the F14 and I20 of ET-1 (Fig. 3b). Overall, while the electrostatic interactions between the carboxylates and the positively charged residues are conserved in IRL2500 and ET-1 binding, the other moieties form distinct interactions with the receptor. The volume of the ligand binding pocket in the ligand-free structure is large, thereby allowing the aromatic moieties of IRL2500 to flip (Fig. 3b).

IRL2500 has distinct chemical moieties as compared with bosentan, because IRL2500 was not developed based on bosentan. To reveal the similarities and differences in their binding modes, we compared the binding modes of IRL2500 and bosentan in detail (Fig. 3e, f). The carboxylate of IRL2500 and the sulfonamide of bosentan are similarly coordinated by the positively charged residue R343^6.55^, suggesting that this electrostatic interaction is a common feature of the antagonist binding to the ET_B_ receptor. In addition, like bosentan, the aromatic moieties of IRL2500 fit within the local hydrophobic pockets in the ET_B_ receptor. Overall, IRL2500 has moieties that form similar binding interactions to those of bosentan. However, bosentan lacks the moiety corresponding to the biphenyl group of IRL2500, which deeply penetrates into the receptor core (Fig. 3f). Thus, IRL2500 fits into the pocket more tightly as compared with bosentan, contributing to its higher affinity for the ET_B_ receptor.

### IRL2500 functions as an inverse agonist

**Fig. 4:**
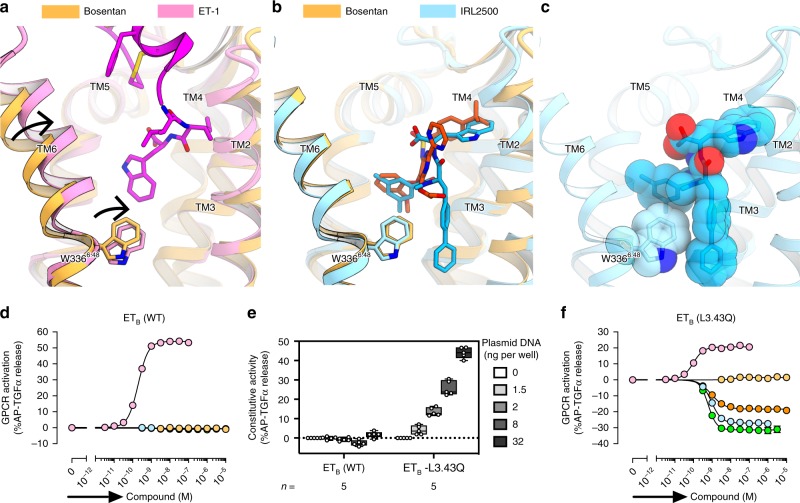
Inverse agonist activity of IRL2500. **a**, **b** Structural changes upon ET-1 and IRL2500 binding, as compared with the bosentan-bound structure, colored as in Fig. 3. Black arrows indicate the inward movements of TM6 and W336^6.48^. **c** CPK representations of IRL2500 and the W336^6.48^ side chain. **d** Effects of ET-1 and the ET_B_ antagonists (bosentan, IRL2500, K-8794, and BQ-788) on the AP-TGFα release for the ET_B_ receptor. For each experiment, the spontaneous AP-TGFα release response in the absence of the compound is set at the baseline. Data are displayed as means ± SEM from six to eleven independent experiments. **e** Constitutive activity of the L192^3.43^Q-mutant ET_B_ receptor (ET_B_-L^3.43^Q). HEK293 cells were transfected with titrated volumes of a plasmid encoding the wild-type ET_B_ (ET_B_-WT) or the L^3.43^Q-mutant ET_B_ (ET_B_-L^3.43^Q) and accumulated AP-TGFα release during 24 h after transfection was measured. The AP-TGFα release signal in the 0 ng receptor plasmid was set as the baseline. Data are displayed as a box-and-whisker plot from five independent experiments with each performed in 4–6 replicates. **f** Effects of ET-1 and the ET_B_ antagonists (bosentan, IRL2500, K-8794 and BQ-788) on the AP-TGFα release for the constitutively active ET_B_ receptor (ET_B_-L^3.43^Q). For each experiment, the spontaneous AP-TGFα release response in the absence of the compound is set at the baseline. Data are displayed as means ± SEM from seven to twelve independent experiments. In the inverse agonist experiments (**d**, **f**), the cells were incubated with a test compound for 4 h

To obtain mechanistic insights into the receptor inactivation by IRL2500, we compared the ET_B_ structures bound to ET-1, bosentan, and IRL2500. Previous structural studies showed that ET-1 binding induces the inward motion of the extracellular portion of TM6 including W336^6.48^, leading to receptor activation on the intracellular side^21^ (Fig. 4a). Bosentan binding sterically prevents the inward motion of W336^6.48^ with its 2-methoxyphenoxy group, and thus functions as an antagonist^22^ (Fig. 4b). The dimethylbenzoyl group of IRL2500 superimposes well with the 2-methoxyphenoxy group of bosentan and similarly prevents the inward motion (Fig. 4b). Moreover, the dimethylbenzoyl and biphenyl groups of IRL2500 sandwich the W336^6.48^ side chain, tightly preventing its inward rotation (Fig. 4c). These observations suggest that IRL2500 tightly prevents the transition to the active state, as compared with bosentan, thereby possibly working as an inverse agonist that reduces the basal activity.

To investigate the inverse agonist activity of IRL2500 for the ET_B_ receptor, we first measured the ligand-induced AP-TGFα release responses. In the wild-type ET_B_, treatment with IRL2500 or the antagonist bosentan with the cells for 4 h-incubation did not change the receptor activation level (Fig. 4d and Table 3). These data suggested that IRL2500 either does not have the inverse agonist activity or that the assay is not sensitive enough to detect the inverse agonist activity. Indeed, we observed that the basal activity of the ET_B_ receptor was very low in the assay and thus we could not distinguish whether IRL2500 functions as an antagonist or an inverse agonist by this assay.

**Table 3:** Characterization of inverse agonist activities of endothelin receptor antagonists

	ET_B_-WT	ET_B_-L192^3.43^Q
**ET-1**		
EC_50_ (nM)	0.19	0.094
pEC_50_ (mean ± SEM)	9.72 ± 0.03	10.03 ± 0.04
*E*_max_ ± SEM	53.6 ± 1.7	20.5 ± 1.3
*n*	10	12
**Bosentan**		
EC_50_ (nM)	NA	NA
pEC_50_ (mean ± SEM)	NA	NA
*E*_max_ ± SEM	<2%	<2%
*n*	11	12
**IRL2500**		
EC_50_ (nM)	NA	0.92
pEC_50_ (mean ± SEM)	NA	9.03 ± 0.05
*E*_max_ ± SEM	<2%	−27.0 ± 1.1
*n*	11	12
**K-8794**		
EC_50_ (nM)	NA	0.61
pEC_50_ (mean ± SEM)	NA	9.21 ± 0.04
*E*_max_ ± SEM	<2%	−31.5 ± 2.0
*n*	6	7
**BQ-788**		
EC_50_ (nM)	NA	0.96
pEC_50_ (mean ± SEM)	NA	9.02 ± 0.03
*E*_max_ ± SEM	<2%	−18.5 ± 0.7
*n*	6	7

Therefore, we tried the same assay by utilizing a mutation to facilitate the constitutive activity of the ET_B_ receptor. Constitutively active mutant GPCRs have been employed in pharmacological characterizations of inverse agonists^30^, because such mutant GPCRs allow the signal measurement in a larger detection window. The substitution of the highly conserved L^3.43^ to glutamine has been identified as a causative activating mutation in the *TSHR*^31^ and *CYSLTR2*^32^ genes, which are related to hyperthyroidism and uveal melanoma, respectively. Therefore, we transferred the L^3.43^Q mutation into the ET_B_ receptor (ET_B_-L192^3.43^Q) and examined its constitutive activity. We found that ET_B_-L^3.43^Q induced spontaneous AP-TGFα release (Fig. 4e), indicating that L^3.43^Q functions as a constitutively active mutation in the ET_B_ receptor. We confirmed that this mutation also increased the constitutive activity in the ET_A_ receptor (Supplementary Fig. 6a). The L^3.43^Q-mutant ET_B_ and ET_A_ increased the potency of both ET-1 (EC_50_) and the antagonists (IC_50_) by approximately 5-fold and 2-fold, respectively (Supplementary Fig. 5b–d). We evaluated the inverse agonist activities of these compounds, using the constitutively active mutant, ET_B_-L^3.43^Q (Fig. 4f and Table 3). As expected, the antagonist bosentan did not change the receptor activation from the baseline level. Conversely, IRL2500 reduced the basal activity of the ET_B_-L^3.43^Q mutant (EC_50_ = 0.92 nM). Both bosentan and IRL2500 did not change the basal activity of the ET_A_-L^3.43^Q (Supplementary Fig. 6b, c). These data indicate that IRL2500 functions as a potent inverse agonist for the ET_B_ receptor, consistent with the structural observations. The biphenyl group of IRL2500 prevents the inward motion of W336^6.48^ to stabilize the inactive conformation, and thus IRL2500 functions as an inverse agonist.

In addition to IRL2500, K-8794 and BQ-788 have been characterized as potent ET_B_-selective antagonists. K-8794 is a high-affinity analog of bosentan^22^, whereas BQ-788 is a peptide analog with distinct chemical moieties, as compared with those of IRL2500 and bosentan^33^. We investigated the inverse agonist activities of K-8794 and BQ-788, using the constitutively active mutants of the ETRs (Fig. 4f and Table 3). K-8794 and BQ-788 reduced the basal activity of the ET_B_-L^3.43^Q with EC_50_ values of 0.61 and 0.96 nM, respectively, indicating that they also function as inverse agonists. K-8794 and BQ-788 showed higher and lower efficacies (*E*_max_) of the inverse agonist activity than that of IRL2500, respectively. The K-8794-bound ET_B_ structure has been determined and thus we compare the ET_B_ structures bound to K-8794, bosentan, and IRL2500. The chemical structure of K-8794 is similar to that of bosentan, but K-8794 has a dimethylphenyl group linked to the peptide bond. This modification slightly displaces the overall position of K-8794, thereby moving W336^6.48^ outward by the interactions with the 6-methoxyphenoxy and the alkyl groups (Supplementary Fig. 7a). Therefore, K-8794 tightly prevents the inward rotation of W336^6.48^, in a similar manner to IRL2500 (Supplementary Fig. 7b). We thus suggest that this structural feature likely contributes to the inverse agonist activity of K-8794.

## Discussion

We have determined the crystal structure of the ET_B_ receptor in complex with the peptide-mimetic drug IRL2500, and thus elucidated the detailed receptor interactions and the structural basis for its ET_B_ selectivity. Although IRL2500 is designed to mimic the partial region of ET-1, its binding mode is quite different. Moreover, using the constitutively active mutant ET_B_-L^3.43^Q, we revealed that IRL2500 together with K-8794 and BQ-788, but not bosentan, function as a potent inverse agonist for the ET_B_ receptor, and provided the structural basis for their inverse agonist activities. Our study sheds light on the new aspects of the ET_B_-selective antagonists, and deepens our understanding of ETR pharmacology.

Although small-molecule ETR antagonists have been developed over the years; however, most ETR antagonists have been designed based on bosentan. Thus, the presently available ETR antagonists are chemically very similar. IRL2500 was developed based on ET-1 and has totally distinct chemical moieties, as compared with those of bosentan. However, the comparison of the IRL2500 and bosentan binding modes revealed the unexpected similarity in their binding interactions. This observation suggests that the charge-complementary interactions in the center of the pocket form the core of the receptor–antagonist interactions, and the other aromatic moieties fit the local hydrophobic pocket. The ligand binding pocket in the inactive ET_B_ structures is larger than those in other GPCR structures, and thus aromatic moieties may be necessary to fit well within the pocket.

**Fig. 5:**
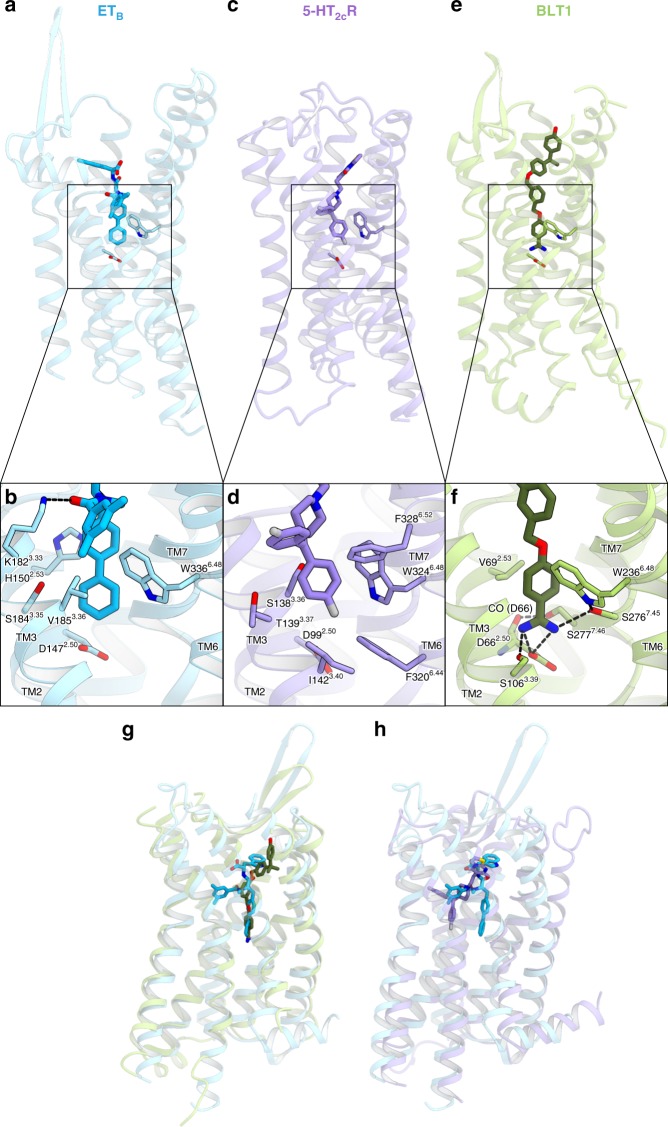
Structural comparison with the inverse agonist-bound GPCR structures. **a**–**f** The structures of the IRL2500-bound ET_B_ receptor (**a**, **b**), Ritanserin-bound 5-HT_2C_R (PDB code: 6BQH) (**c**, **d**), and the BIIL260-bound BLT1 (PDB code: 5X33) (**e**, **f**), shown as sky blue, purple and light green ribbons, respectively. The inverse agonists IRL2500, ritanserin, and BIIL260 are shown as sticks. The binding interactions around the sodium binding site are shown in (**b**), (**d**), and (**f**). The residues involved in ligand binding are represented with sticks. Hydrogen bonds are indicated by black dashed lines. In the IRL2500-bound ET_B_ structure, the biphenyl group of IRL2500 forms van der Waals interactions with the receptor, and does not form any hydrogen-binding interactions. In the BIIL260-bound BLT1 structure, BIIL260 forms hydrogen bonds with D66^2.50^, S106^3.39^ and S276^7.45^. **g**, **h** Superimposition of the IRL2500-bound ET_B_ structure with the BLT1 (**g**) and 5-HT_2C_R (**h**) structures

Our study revealed that the biphenyl group of IRL2500 penetrates deeply into the receptor core proximal to D147^2.50^, preventing the inward motion of W336^6.48^ in TM6, and thus IRL2500 functions as an inverse agonist (Fig. 5a, b). The deep binding modes of the inverse agonists are also observed in the 5-HT_2C_R and BLT1 structures (Fig. 5c–f). In the 5-HT_2C_R structure, the 4-fluorophenyl group of the inverse agonist ritanserin interacts with F320^6.44^ and W324^6.48^, the purported “toggle switch” important for GPCR activation^34^ (Fig. 5c, d). In the BLT1 structure, the benzamidine group of the inverse agonist BIIL260 fits into a sodium binding pocket around D147^2.50^ (Fig. 5e, f), which is highly conserved among the class A GPCRs^35^. Sodium selectively competes with agonist binding in most class A GPCRs by stabilizing the inactive conformations, and the sodium binding site is thus an important pocket targeted in the design of negative allosteric modulators and inverse agonists. Instead of the sodium, the benzamidine group of BIIL260 directly hydrogen bonds with D^2.50^, similarly stabilizing the inactive conformation^36^ (Fig. 5f). The binding mode of IRL2500 is similar to that of BIIL260 in BLT1, rather than that of ritanserin in the 5-HT_2C_R (Fig. 5g, h). Although the biphenyl group of IRL2500 does not form any hydrogen-bonding interactions with the receptor, it prevents the conformational change around the D2.50 in a similar manner to the benzamidine moiety of BIIL260 (Fig. 5b, f). For the design of effective inverse agonists, the biphenyl moiety would also be useful as a modulation part along with another moiety that exerts specific and tight binding to the orthosteric site, as well as a benzamidine group.

## Methods

### Expression and purification

The haemagglutinin signal peptide, followed by the Flag epitope tag (DYKDDDDK) and a nine-amino-acid linker, was added to the N-terminus of the receptor, and a tobacco etch virus (TEV) protease recognition sequence was introduced between G57 and L66, to remove the disordered N-terminus during the purification process. The C-terminus was truncated after S407, and three cysteine residues were mutated to alanine (C396A, C400A, and C405A) to avoid heterogeneous palmitoylation. To improve crystallogenesis, we introduced four thermostabilizing mutations (R124Y^1.55^, K270A^5.35^, S342A^6.54^, and I381A^7.48^) and inserted minimal T4 lysozyme^25^ into intracellular loop 3, between K303^5.68^ and L311^6.23^ (ET_B_-Y4-mT4L^22^).

The thermostabilized construct ET_B_-Y4-mT4L was subcloned into a modified pFastBac vector, with the resulting construct encoding a TEV cleavage site followed by a GFP-His^10^ tag at the C-terminus. The recombinant baculovirus was prepared using the Bac-to-Bac baculovirus expression system (Invitrogen). Sf9 insect cells were infected with the virus at a cell density of 4.0 × 10^6^ cells per ml in Sf900 II medium, and grown for 48 h at 27 °C. The harvested cells were disrupted by sonication, in buffer containing 20 mM Tris–HCl, pH 7.5, and 20% glycerol. The crude membrane fraction was collected by ultracentrifugation at 180,000 *g* for 1 h. The membrane fraction was solubilized in buffer, containing 20 mM Tris–HCl, pH 7.5, 200 mM NaCl, 1% DDM, 0.2% cholesterol hemisuccinate, 10 μM IRL2500, and 2 mg ml^−1^ iodoacetamide, for 1 h at 4 °C. The supernatant was separated from the insoluble material by ultracentrifugation at 180,000 *g* for 20 min, and incubated with TALON resin (Clontech) for 30 min. The resin was washed with ten column volumes of buffer, containing 20 mM Tris–HCl, pH 7.5, 500 mM NaCl, 0.1% LMNG, 0.01% CHS, 10 μM IRL2500, and 15 mM imidazole. The receptor was eluted in buffer, containing 20 mM Tris–HCl, pH 7.5, 500 mM NaCl, 0.01% LMNG, 0.001% CHS, 10 μM IRL2500, and 200 mM imidazole. The eluate was treated with TEV protease and dialyzed against buffer (20 mM Tris–HCl, pH 7.5, 500 mM NaCl, and 10 μM IRL2500). The cleaved GFP-His_10_ tag and the TEV protease were removed with Co^2+^-NTA resin. The receptor was concentrated and loaded onto a Superdex200 10/300 Increase size-exclusion column, equilibrated in buffer containing 20 mM Tris–HCl, pH 7.5, 150 mM NaCl, 0.01% LMNG, 0.001% CHS, and 10 μM IRL2500. Peak fractions were pooled, concentrated to 40 mg ml^−1^ using a centrifugal filter device (Millipore 50 kDa MW cutoff), and frozen until crystallization. During the concentration, IRL2500 was added to a final concentration of 100 μM.

### Crystallization

The purified receptor was reconstituted into molten lipid (monoolein and cholesterol 10:1 by mass) at a weight ratio of 1:1.5 (protein:lipid)^37^. The protein-laden mesophase was dispensed into 96-well glass plates in 30 nl drops and overlaid with 800 nl precipitant solution by a Gryphon LCP robot (Art Robbins Instruments)^26^. Crystals of ET_B_-Y4-mT4L bound to IRL2500 were grown at 20 °C in precipitant conditions containing 30% PEG300, 100 mM Bis–tris, pH 7.5, 150 mM sodium phosphate monobasic, and 10 mM TCEP hydrochloride. The crystals were harvested directly from the LCP using micromounts (MiTeGen) or LithoLoops (Protein Wave) and frozen in liquid nitrogen, without adding any extra cryoprotectant.

### Data collection and structure determination

X-ray diffraction data were collected at the SPring-8 beamline BL32XU, with 10 × 15 μm^2^ (width × height) micro-focused beams and an EIGER X 9M detector (Dectris). Various wedge data sets (10°) per crystal were mainly collected with the ZOO system^38^, an automatic data-collection system developed at SPring-8. The loop-harvested microcrystals were identified by raster scanning and subsequently analyzed by SHIKA^39^. The collected images were automatically processed with KAMO^40^ (https://github.com/keitaroyam/yamtbx). Each data set was indexed and integrated with XDS^41^, and the datasets were hierarchically clustered by using the correlation coefficients of the intensities between datasets^41^. After the rejection of outliers, 58 data sets were finally merged with XSCALE^41^. The IRL2500-bound structure was determined by molecular replacement with PHASER^42^, using the K-8794-bound ET_B_ structure (PDB code: 5X93). Subsequently, the model was rebuilt and refined using COOT^43^ and PHENIX^44^, respectively. The final model of IRL2500-bound ET_B_-Y4-T4L contained residues 91-207, 214-303, and 311-403 of ET_B_, 1-11 and 19-117 of mT4L, IRL2500, 8 monoolein molecules, 4 phosphoric acids, and 34 water molecules. The model quality was assessed by MolProbity^45^. Figures were prepared using CueMol (http://www.cuemol.org/ja/).

### TGFα shedding assay

The TGFα shedding assay, which measures the activation of G_q/11_ and G_12/13_ signaling^24^, was performed as described previously^22^. Briefly, a plasmid encoding an ET_B_ construct with an internal FLAG epitope tag or an ET_A_ construct was transfected, together with a plasmid encoding alkaline phosphatase (AP)-tagged TGFα (AP-TGFα), into HEK293A cells by using a polyethylenimine (PEI) transfection reagent (1 µg ETR plasmid, 2.5 µg AP-TGFα plasmid, and 25 µl of 1 mg per ml PEI solution per 10-cm culture dish). After a 1-day culture, the transfected cells were harvested by trypsinization, washed, and resuspended in 30 ml of Hank’s Balanced Salt Solution (HBSS) containing 5 mM HEPES (pH 7.4). The cell suspension was seeded in a 96-well plate (cell plate) at a volume of 80 μl per well and incubated for 30 min in a CO_2_ incubator. For the measurement of antagonist activity, IRL2500 was diluted in 0.01% bovine serum albumin (BSA) and HEPES-containing HBSS (assay buffer) and added to the cell plate at a volume of 10 µl per well. After 5 min, ET-1, at a final concentration of 0.5 nM, was added to the cell plate at a volume of 10 µl per well. For the measurement of agonistic activity, after adding 10 µl of the assay buffer, serially diluted ET-1 was mixed with the cells at a volume of 10 µl per well. After a 1 h incubation in the CO_2_ incubator, aliquots of the conditioned media (80 μl) were transferred to an empty 96-well plate (conditioned media (CM) plate). Similarly, for the measurement of inverse agonist activity, the cells were mixed with 10 µl of the assay buffer, followed by the addition of serially diluted IRL2500, and incubated for 4 h before the transfer of the conditioned media. The AP reaction solution (10 mM *p*-nitrophenylphosphate (*p*-NPP), 120 mM Tris–HCl (pH 9.5), 40 mM NaCl, and 10 mM MgCl_2_) was dispensed into the cell plates and the CM plates (80 µl per well). The absorbance at 405 nm (Abs_405_) of the plates was measured, using a microplate reader (SpectraMax 340 PC384, Molecular Devices), before and after a 1 h incubation at room temperature. AP-TGFα release was calculated as described previously^22^. The AP-TGFα release signals were fitted to a four-parameter sigmoidal concentration-response curve, using the Prism 7 software (GraphPad Prism), and the pEC_50_ (equal to −log_10_ EC_50_) and *E*_max_ values were obtained.

To obtain equilibrium dissociation constant (*K*_B_), for each experiment performed in parallel, IC_50_ values (IRL2500, K-8794, BQ-788), an EC_50_ value (ET-1), a Hill slope (*K*, ET-1), and tested concentration of ET-1 (*A*; 0.5 nM) were processed as follows^46^:

$$K_{\mathrm{B}} = \frac{{\mathrm{IC}_{50}}}{{1 + \left( {\frac{A}{{\mathrm{EC}_{50}}}} \right)^K}}$$

The resulting *K*_B_ values were logarithmically transformed and their negative values (p*K*_B_) were used to calculate a difference in p*K*_B_ value (∆p*K*_B_) for a mutant (MT) receptor as follows by using p*K*_B_ value for WT receptor performed in parallel:

$$\Delta \mathrm{p}K_{\mathrm{B}} = \mathrm{p}K_{{\mathrm{B}}\left( {{\mathrm{MT}}} \right)} - \mathrm{p}K_{{\mathrm{B}}\left( {{\mathrm{WT}}} \right)}$$

The p*K*_B_ and the ∆p*K*_B_ values were used to calculate mean and SEM.

To measure the constitutive activity in a plasmid volume-dependent manner, HEK293 cells were seeded in a 96-well plate at a concentration of 4 × 10^5^ cells per ml in Opti-MEM I Reduced Serum Media (Thermo Fisher Scientific), in a volume of 80 μl per well. A transfection mixture was prepared by mixing the PEI transfection reagent (0.2 µl per well) and plasmids (20 ng AP-TGFα plasmid, titrated ETR plasmid, and an empty vector to balance the total plasmid volume) in Opti-MEM I Reduced Serum Media (20 µl). The mixture was added to the cells, which were then incubated for 24 h before the transfer of the conditioned media. After adding the AP reaction solution, the absorbances of the cells and the CM plates were measured at 20 min intervals. The AP-TGFα release signals were calculated as described above, and the signal in the mock-transfected conditions was set at the baseline. A glutamine mutation was introduced into the internal FLAG epitope-tagged ET_B_ and the ET_A_ constructs at L192^3.43^ and L176^3.43^, respectively.

### Reporting summary

Further information on research design is available in the Nature Research Reporting Summary linked to this article.

## Supplementary information

Supplementary Information

Reporting Summary

## Data Availability

Coordinates and structure factors have been deposited in the Protein Data Bank, under the accession number 6K1Q for the IRL2500-bound structure. The raw X-ray diffraction images are also available at Zenodo (https://zenodo.org/record/2803553). All other data are available from the authors upon reasonable request.
