# Supplementary material for: Crystal structure of human endothelin ETB receptor in complex with peptide inverse agonist IRL2500
Source: Commun Biol. 2019 Jun 21;2:236. doi: 10.1038/s42003-019-0482-7 (PMC6588608; doi:10.1038/s42003-019-0482-7)
Supplement: Supplementary file 1 — Supplementary Information [file 42003_2019_482_MOESM1_ESM.pdf]

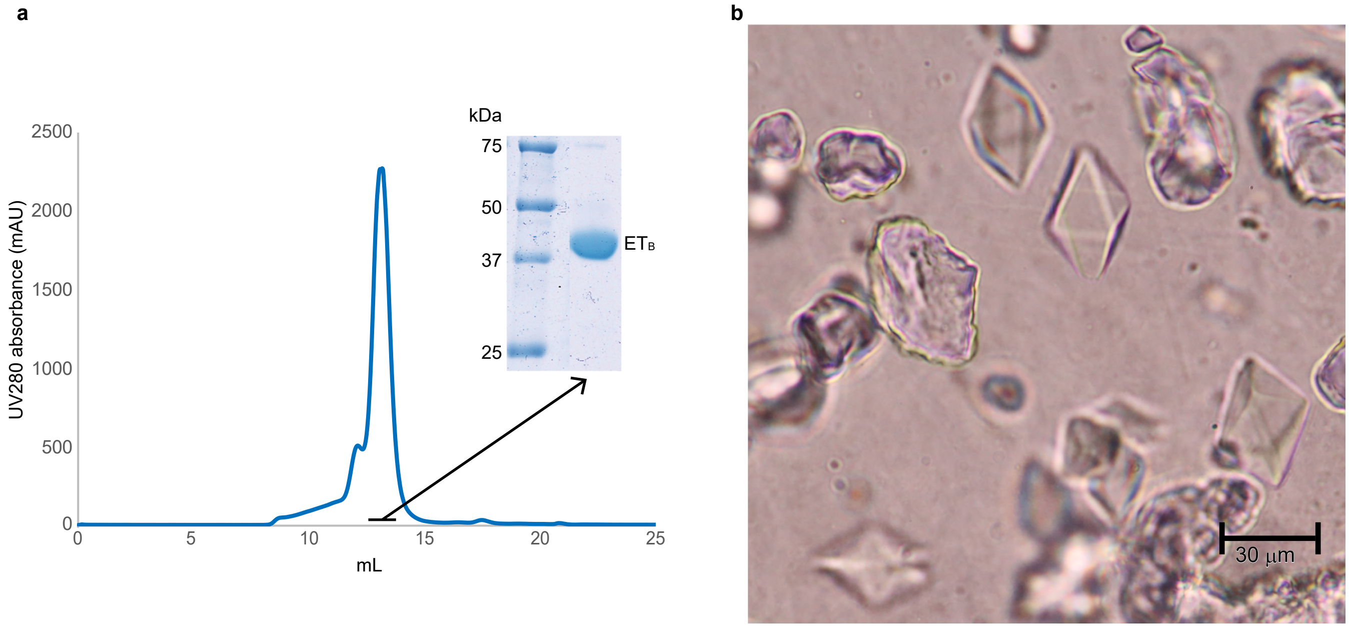

Supplementary Figure 1 | Crystallization.
a, Gel filtration chromatogram and SDS-PAGE of the purified IRL2500-bound ET_B receptor. **b,** Crystals of the IRL2500-bound ET_B receptor.

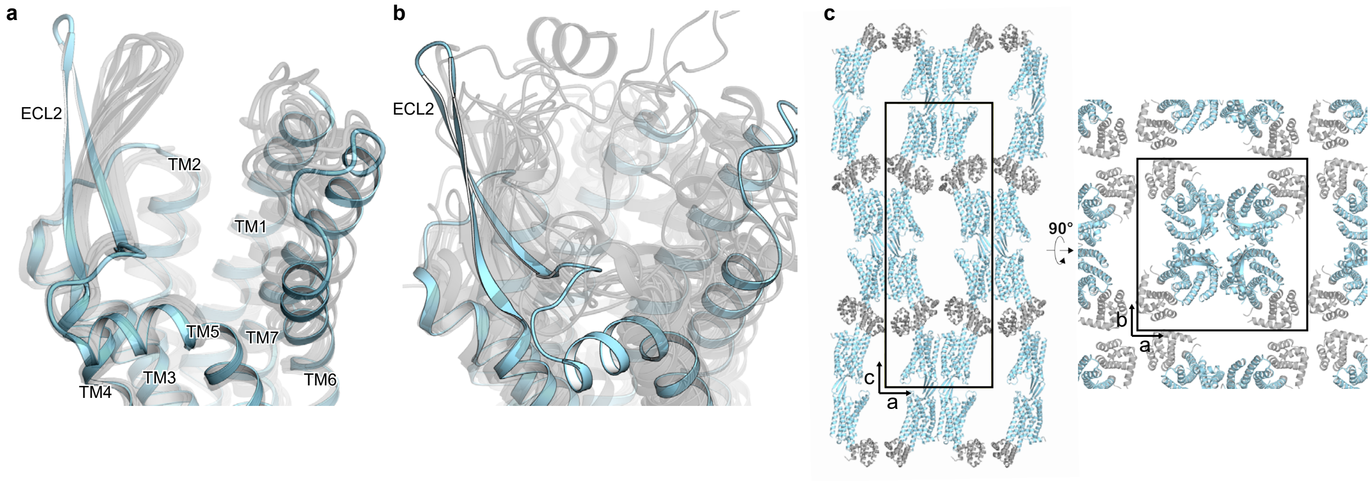

Supplementary Figure 2 | Comparison of the ECL2 structure with those of other peptide-activated GPCRs.
a, Superimposition of the ET_B structures determined to date. The IRL2500-bound and other structures are coloured sky blue and gray, respectively. **b,** Superimposition of the IRL2500-bound ET_B structure with other peptide-activated class A GPCRs. **c,** Crystal packing of the IRL2500-bound ET_B structure. The receptor and T4L are shown as grey and blue ribbons, respectively. The crystal lattice is indicated by black lines.

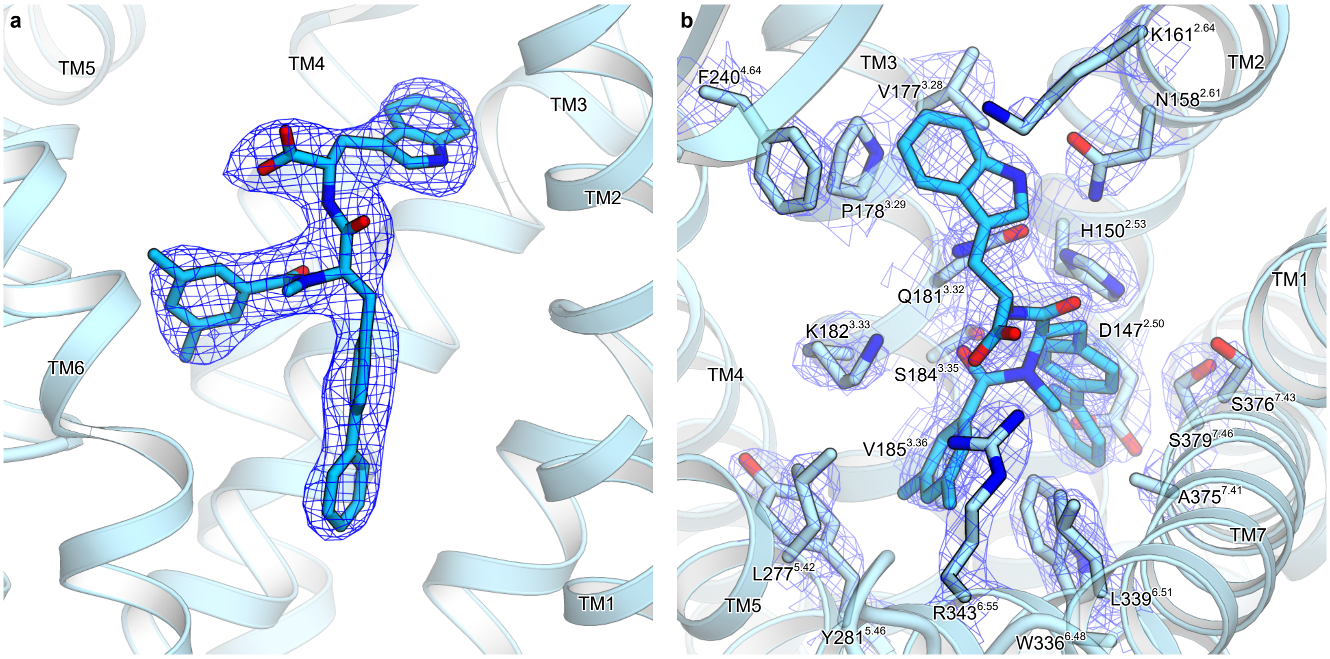

Supplementary Figure 3 | Electron density.

a, $F_o - F_c$ omit maps for IRL2500, contoured at 3.0σ. **b,** $2F_o - F_c$ map around the IRL2500 binding site, contoured at 2.5σ.

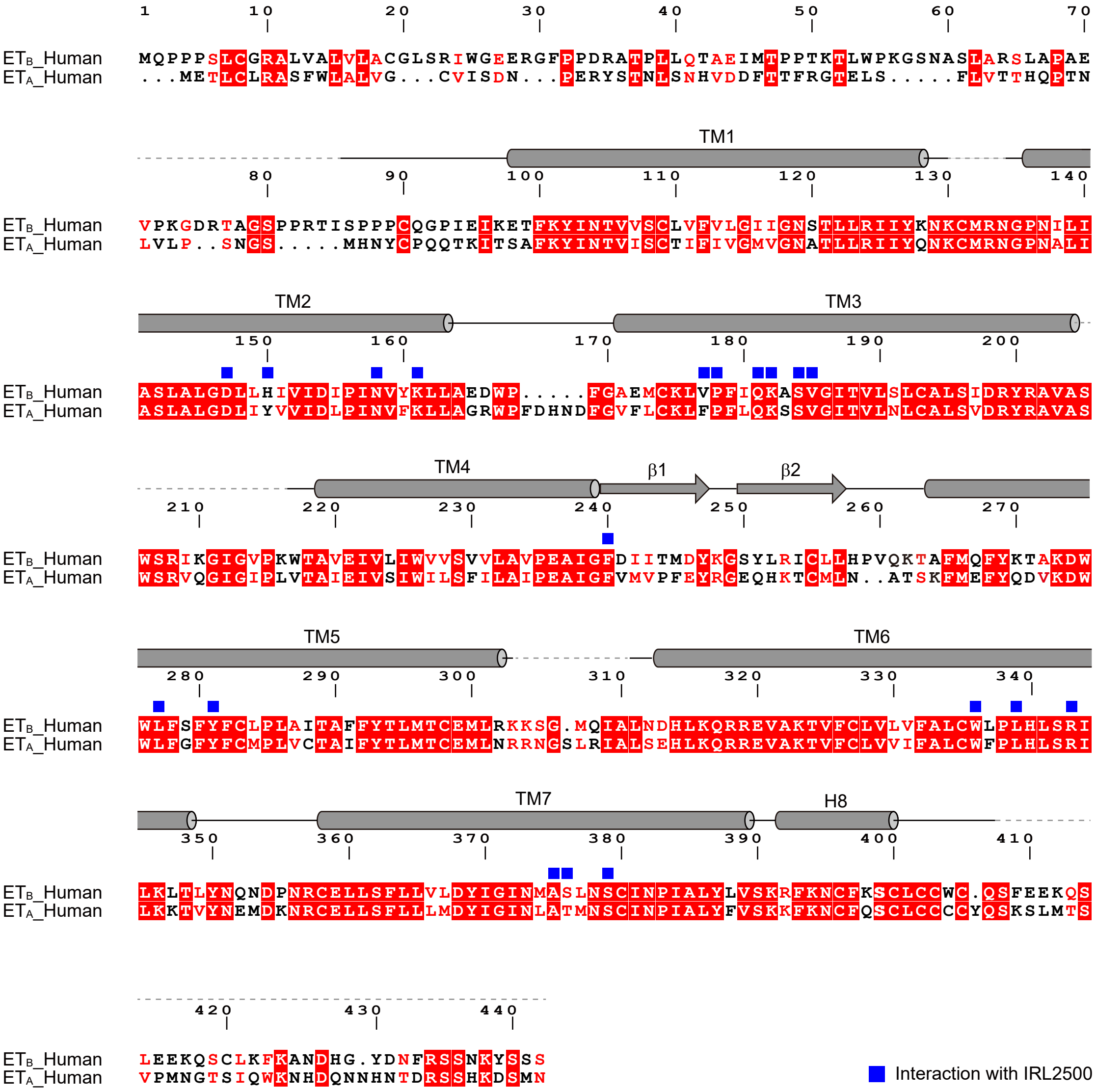

Supplementary Figure 4 | Alignment of the human ET$_A$ and ET$_B$ receptors.
Alignment of the amino acid sequences of the human ET$_B$ receptor (Uniprot ID: P24530) and the human ET$_A$ receptor (P25101). Secondary structure elements for α-helices and β-strands are indicated by cylinders and arrows, respectively. Conservation of the residues between ET$_A$ and ET$_B$ is indicated as follows: red panels for completely conserved; red letters for partially conserved; and black letters for not conserved. The residues involved in IRL2500 binding are indicated with squares.

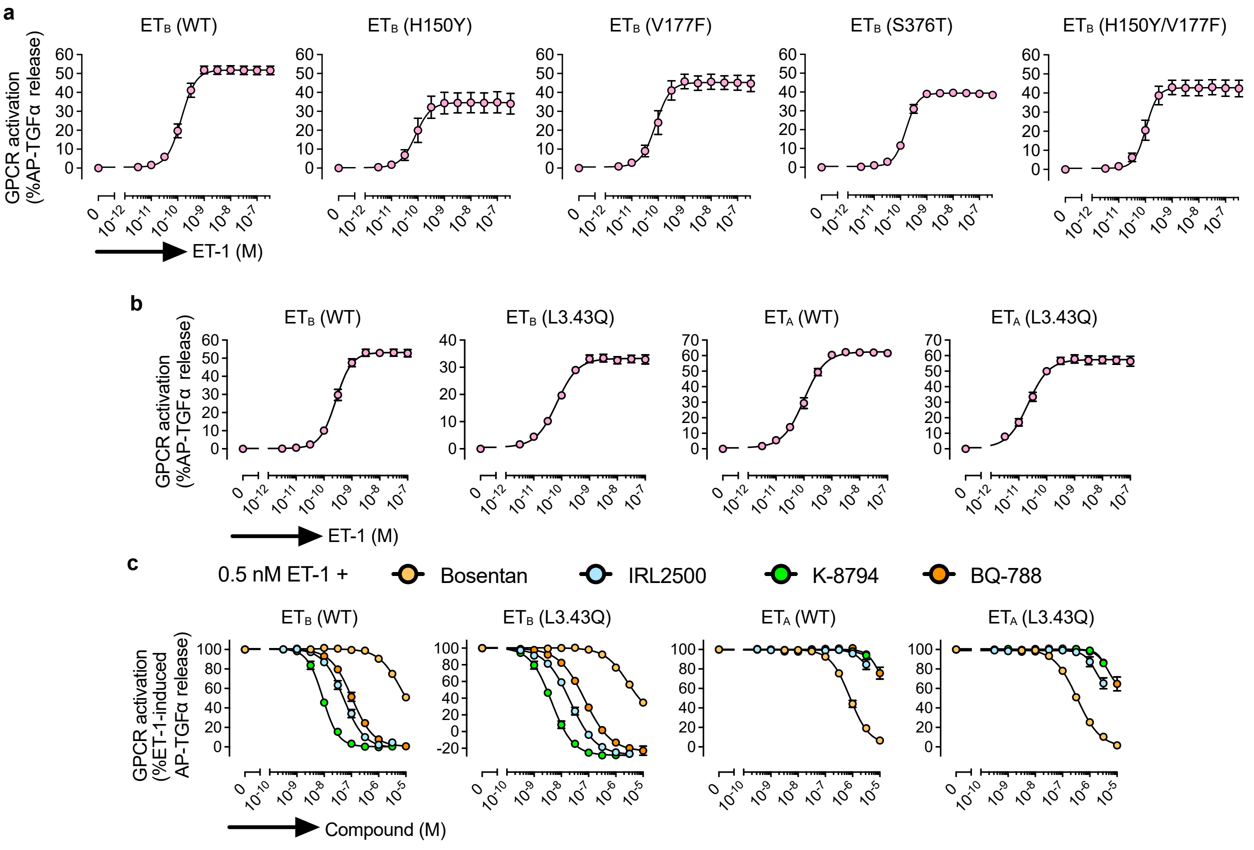

d

		ET_B (WT)	ET_B (L3.43Q)	ET_A (WT)	ET_A (L3.43Q)
ET-1	*n* =	6	7	7	5
	EC_{50} (pEC_{50} ± SEM)	0.27 nM (9.57 ± 0.06)	0.069 nM (10.16 ± 0.08)	0.10 nM (9.98 ± 0.09)	0.021 nM (10.67 ± 0.07)
Bosentan	*n* =	6	7	7	5
	IC_{50} (pIC_{50} ± SEM)	9800 nM (5.01 ± 0.04)	4400 nM (5.36 ± 0.06)	850 nM (6.07 ± 0.06)	340 nM (6.47 ± 0.03)
IRL2500	*n* =	5	6	6	5
	IC_{50} (pIC_{50} ± SEM)	52 nM (7.28 ± 0.07)	22 nM (7.67 ± 0.06)	>3 μM	>3 μM
K-8794	*n* =	6	7	7	5
	IC_{50} (pIC_{50} ± SEM)	8.7 nM (8.06 ± 0.04)	4.2 nM (8.37 ± 0.06)	>3 μM	>3 μM
BQ-788	*n* =	6	7	7	5
	IC_{50} (pIC_{50} ± SEM)	110 nM (6.97 ± 0.07)	75 nM (7.13 ± 0.08)	>10 μM	>10 μM

Supplementary Figure 5 | Phamacological characterization of mutant receptors.
a, b, Concentration response-curves of AP-TGFα release in the ET-1 treatment of HEK293 cells expressing the mutant ET_B receptors (**a**) and the constitutively active L3.43Q-mutant receptors (**b**). Symbols and error bars are means and s.e.m. (standard error of the mean) of four to seven independent experiments, each performed in duplicate or triplicate. **c,** Effect of the antagonists on ET-1 (0.5 nM)-induced responses of AP-TGFα release in HEK293 cells expressing the wild-type or constitutively active endothelin receptors. For each experiment, the AP-TGFα release response in the absence of the antagonist is set at 100%. Data are displayed as means ± s.e.m. (standard error of the mean) from five to seven independent experiments, with each performed in duplicate or triplicate. **d,** Parameters for the agonist and the antagonists. From the concentration-response curves in (**b**) and (**c**), pEC_{50} values (ET-1) and pIC_{50} values (antagonists) were calculated. Mean pEC_{50} and pIC_{50} values were used to calculate EC_{50} and IC_{50} values, respectively.

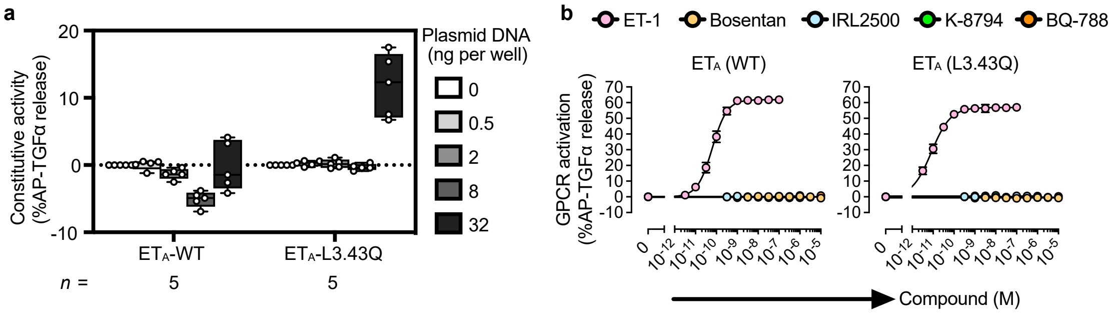

c

		ET_A (WT)	ET_A (L3.43Q)
ET-1	*n* =	6	5
	EC_{50}, (pEC_{50} ± SEM)	0.064 nM, (10.20 ± 0.09)	0.0082 nM, (11.09 ± 0.09)
	E_{max}	62.0 ± 1.6	56.8 ± 2.1
Bosentan	*n* =	8	5
	EC_{50}, (pEC_{50} ± SEM)	NA	NA
	E_{max}	<2%	<2%
IRL2500	*n* =	6	5
	EC_{50}, (pEC_{50} ± SEM)	NA	NA
	E_{max}	<2%	<2%
K-8794	*n* =	7	5
	EC_{50}, (pEC_{50} ± SEM)	NA	NA
	E_{max}	<2%	<2%
BQ-788	*n* =	7	5
	EC_{50}, (pEC_{50} ± SEM)	NA	NA
	E_{max}	<2%	<2%

Supplementary Figure 6 | Characterization of inverse agonist activities for the ET_A receptors.
a, Constitutive activity of the L3.43Q-mutant ET_A receptors (ET_A-L3.43Q). HEK293 cells were transfected with a titrated volume of a plasmid encoding the wildtype ET_A (ET_A-WT) or the ET_A-L3.43Q and the accumulated AP-TGFα release during 24 h after transfection was measured. The AP-TGFα release signal in the 0 ng receptor plasmid was set as the baseline. **b, c,** Effects of ET-1 and the ET_B antagonists (bosentan, IRL2500, K-8794 and BQ-788) on the AP-TGFα release for the wild-type or constitutively active ET_A receptor (ET_A-L3.43Q). The cells were incubated with the compounds for 4 h, instead of 1 h, the condition that was used for the other antagonist experiments. For each experiment, the AP-TGFα release response in the absence of the compound is set at the baseline. Data are displayed as means ± s.e.m. from five to eight independent experiments.

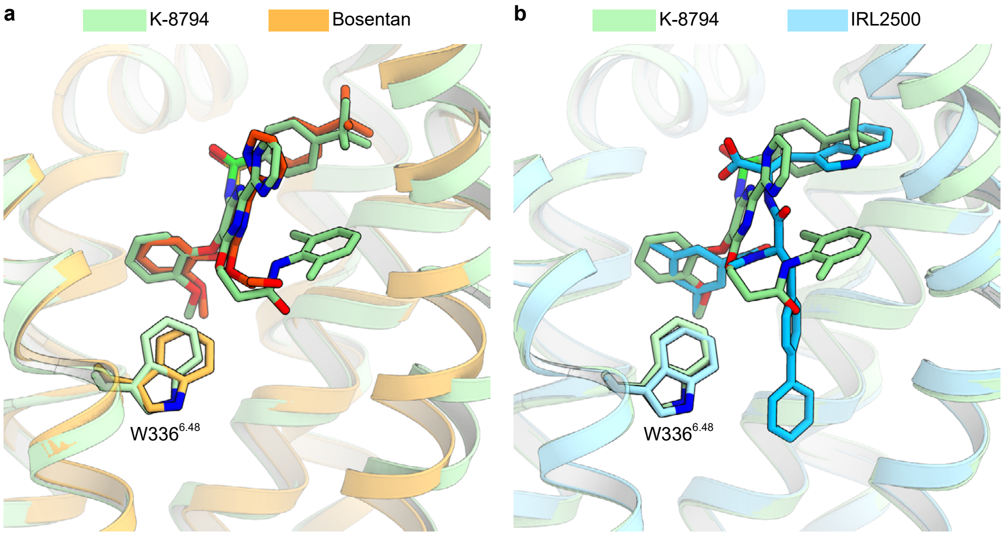

Supplementary Figure 7 | Comparison of the K-8794, bosentan, and IRL2500-bound structures.
a,b, Superimpositions of the K-8794-bound receptor with the bosentan- (**a**) and IRL2500-bound receptors (**b**).
